# Classification of central facial paralysis: an agreement analysis

**DOI:** 10.1590/2317-1782/e20240158en

**Published:** 2025-05-02

**Authors:** Nathallie Angel Conceição da Silva Andrade, Raquel Karoline Gonçalves Amaral, Laélia Cristina Caseiro Vicente, Aline Mansueto Mourão

**Affiliations:** 1 Departamento de Fonoaudiologia, Universidade Federal de Minas Gerais – UFMG - Belo Horizonte (MG), Brasil.

**Keywords:** Facial Paralysis, Evaluation, Scales, Classification, Speech Therapy

## Abstract

**Purpose:**

To identify whether there is interrater and intrarater agreement in the classification of the degree of central facial paralysis using two scales and verify which one is more appropriate to classify the severity of facial expressions according to experts’ opinion.

**Method:**

Observational, prospective, cross-sectional study of agreement analysis of the House & Brackmann (HB) scale and the Sunnybrook Facial Grading System (SFGS). Five speech-language-hearing pathologists with clinical experience analyzed post-stroke facial expression of 30 adults for interrater agreement. They were evaluated in two stages, with a 10-day interval; the second stage involved 20% ​​of the initial sample for intrarater agreement. The study used weighted kappa coefficient for the HB scale and the intraclass correlation coefficient for the SFGS classification.

**Results:**

The HB scale indicated considerable interrater and excellent intrarater agreements. The SFGS had good interrater and intrarater agreements. All speech-language-hearing pathologists considered the SFGS the most appropriate scale for classifying central facial paralysis.

**Conclusion:**

The SFGS performed better in interrater agreement analysis. The HB scale had considerable merits in the intrarater assessment. Both scales are adaptable and useful to assess and classify central facial paralysis. However, the speech-language-hearing pathologists indicated the SFGS as the most appropriate.

## INTRODUCTION

Central facial paralysis (CFP) is one of the most common consequences in patients after a stroke, affecting more than half of them^(1)^. This condition causes facial asymmetry and muscular incapacity, mainly for facial movements in the lower face, influencing the patient’s functioning, aesthetic, social, and emotional aspects^(1,2)^.

CFP differs from peripheral facial paralysis (PFP) mainly in the location of the lesion and the type of movements and third of the face affected. PFP involves voluntary and involuntary movements in the entire hemiface ipsilateral to the lesion and is caused by lesions of the facial nerve (infranuclear innervation) or the nucleus of the facial nerve (nuclear). CFP lesion, in turn, occurs above the nucleus of the facial nerve in the brainstem, usually due to lesions or dysfunctions in the cerebral cortex, in the corticobulbar pathway or adjacent areas. In this case, it affects the voluntary muscles of the lower and midface contralateral to the lesion, due to the nucleus of the facial nerve that innervates the lower hemiface and receives corticonuclear fibers (supranuclear innervation) from the contralateral hemisphere. The facial nucleus that innervates the upper segment of the face receives corticonuclear fibers from both cerebral hemispheres, preserving facial expressions of the upper hemiface in lesions of the corticonuclear tract^(2)^.

It is important to note that PFP usually results from a stroke in the middle cerebral artery or its branches. However, strokes in other arteries, such as the basilar or middle meningeal arteries, can also cause PFP, as they help irrigate the facial nerve^(3)^.

Several protocols in the literature classify PFP, including the House & Brackmann (HB) (1985)^(4)^, Chevalier (1987)^(5)^, and Yahanagihara (1976)^(6)^ scales, and the Sunnybrook Facial Grading System (SFGS) (1994)^(7)^. However, research did not find exclusive scales for classifying facial expressions in CFP. The HB scale evaluates the three thirds of the face at rest and in movement, and its classification consists of six degrees. According to the literature, it is the most used in both types of paralysis in clinical practice and scientific research^(8)^, being adapted to CFP cases^(9)^. The SFGS analyzes the face in three clinical parameters: symmetry at rest, symmetry in movement, and the occurrence of contractures and synkineses. Its main objective is to classify paralysis based on facial functioning^(7)^.

In health professionals’ experience, assessment is fundamental in identifying and defining the patient's clinical conditions and the resources available to determine the appropriate intervention for functional performance^(10)^. Being able to analyze abnormal movements correctly is essential for evaluating patients with functionally impaired movements^(10)^.

Hence, using specific scales to measure the degree of CFP can have a major impact on prioritizing interventions and monitoring patient progress^(11)^. It is extremely important in clinical speech-language-hearing (SLH) practice to use scales with a high degree of reliability and trustworthiness to improve the quality of care and optimize the professionals' time^(11)^. Therefore, CFP may require a more targeted assessment, requiring the professional to pay specific attention to abnormal movements that compromise facial expressions. Therefore, verifying whether the scales validated for PFP can be applied to CFP is important for clinical SLH practice because it corroborates the correct diagnosis and feasible intervention based on the patient's functional performance.

Thus, this study aimed to identify 1) whether there is interrater and intrarater agreement in the classification of the degree of CFP, using two scales (HB and SFGS) and 2) whether either of these is more appropriate for CFP according to experts’ opinion.

## METHODS

This cross-sectional observational study was approved by the Research Ethics Committee of the Federal University of Minas Gerais under evaluation report no. 5.019.519. The study invited five SLH pathologists with experience in facial paralysis rehabilitation to evaluate 30 clinical cases of patients with post-stroke CFP. They agreed to participate in the research and signed an informed consent form.

Their evaluation was based on photographs and videos collected from the healthcare database of patients with post-stroke CFP at the Stroke Unit of the Risoleta Tolentino Neves Hospital. They contained the following facial expressions: “at rest”, “scared face”, “angry face”, “close eyes gently”, “close eyes tightly”, “open smile”, “closed smile”, “sad face”, “bad smell”, “pouting”, and “pursed lips”.

The five SLH pathologists evaluated each patient’s photographs and videos using two different scales: HB (1985) (4), which has not been adapted or validated for Brazilian Portuguese, and SFGS (1994) (7), which was translated, adapted, and validated for Brazilian Portuguese in 2022^(12)^.

The HB scale (Chart 1) assesses facial expressions at rest and in movement, classifying facial paralysis according to the level of paralysis affected by the thirds of the face, graded into six levels^(4)^. The adapted HB scale assesses CFP patients at rest (analyzing facial symmetry in the three thirds of the face) and in movement (approaching only the lower face)^(4)^.

**Chart 1:** Facial movement assessment according to House & Brackmann (1985)

**Degree**	**Description**	**At rest**	**In movement**
I **( )**	Normal	Symmetry	Normal facial function
II **( )**	Mild dysfunction	Normal symmetry and tone	*Forehead*: moderate to good function
			*Eyes*: complete closure with minimal effort
			*Mouth*: slight asymmetry
III **( )**	Moderate dysfunction	Normal symmetry and tone	*Forehead*: slight to moderate movement
			*Eyes*: complete closure with effort
			*Mouth*: slight weakness with maximum effort
IV **( )**	Moderately severe dysfunction	Normal symmetry and tone	*Forehead*: none
			*Eyes*: incomplete closure
			*Mouth*: asymmetry with maximum effort
V **( )**	Severe dysfunction	Asymmetry	*Front*: none
			*Eyes*: incomplete closure
			*Mouth*: slight movement
VI **( )**	Total paralysis	Asymmetry	No movement

**Figure 1:**
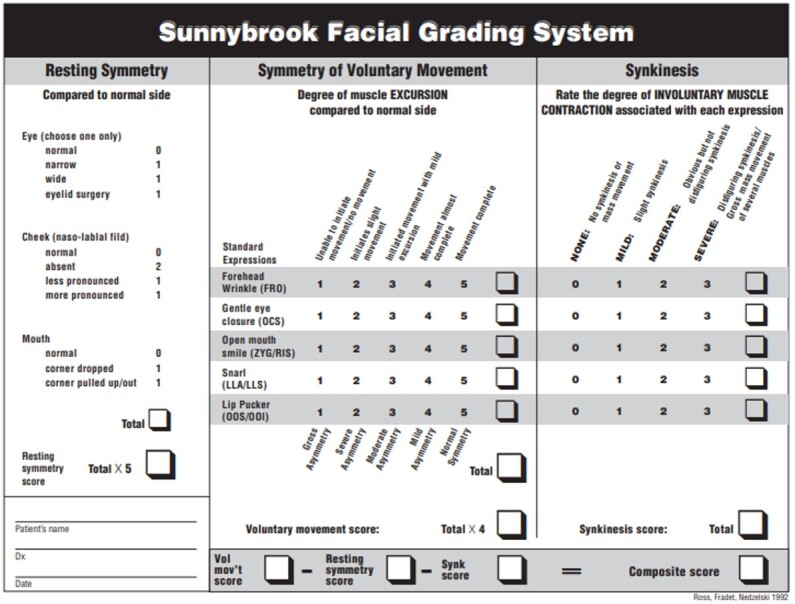
Sunnybrook Facial Grading System (2022)

The SFGS (Figure 1) assesses facial expressions regarding symmetry at rest and of voluntary movements and considers synkineses with the movements tested^(7,12)^. The main differences between the scales are that the SFGS offers a broader and more detailed numerical result, considering the function of specific facial muscles, and verify the occurrence of synkineses.

The SLH pathologists participating in the study did not habitually use the HB and SFGS scales, although they had experience in monitoring patients with facial paralysis. Therefore, they were initially trained online to set a standard technical basis to assess facial paralysis with the two scales. The training presented the scales and clarified the forms of assessment, as they are not specific to CFP, and professionals could use them in different ways, making the results agreement analysis and the study unfeasible. Hence, the training equipped professionals with tools on how to use the scales and not how to interpret facial expressions.

The training used three CFP cases other than the 30 cases presented for analysis. The latter were selected from the database of photos and videos through stratified sampling according to the HB classification performed by an experienced SLH pathologist to balance the severity levels of the paralysis.

After training, the SLH pathologists received photographs and videos of the 30 clinical cases for interrater agreement. They were given 20 days to evaluate the cases as many times as necessary using the two scales (HB and SFGS), but they could not discuss them with peers.

The process of researching intrarater agreement began through the second assessment, 10 days after they submitted the first one. To this end, the five SLH pathologists received 20% of the initial sample (six cases), categorized differently from the first assessment, so that they could re-evaluate them using the same criteria established in the first assessment, likewise using the two scales.

In addition to evaluating the cases, the SLH pathologists were asked to answer the Facial Paralysis Classification Preference Questionnaire (Chart 2), developed by the authors with two questions, one defining the best scale for this assessment and the other explaining the reasons for the choice.

**Chart 2:** Facial Paralysis Classification Preference Questionnaire

1. Which scale is the most appropriate for assessing central facial paralysis?
( ) House & Brackmann (HB)
( ) Sunnybrook Facial Grading System (SFGS)
2. Please, share the reason(s) for your choice

The SLH pathologists' responses were tabulated, and the results were subjected to statistical analysis using SPSS, version 20. The interrater and intrarater agreement analyses were performed with the weighted kappa coefficient (K) for the HB classification (as it is an ordinal categorical variable) and the intraclass correlation coefficient (ICC) for the SFGS classification (as it is measured as a continuous variable). The analyses measured the agreement between two or more raters^(13,14)^. The intrarater agreement was verified considering the first and second assessments with a reproducibility of 20% of the total sample.

Kappa values ​​equal to 0 were considered as poor agreement, from 0 to 0.20 as slight, from 0.21 to 0.40 as considerable, from 0.41 to 0.60 as moderate, from 0.61 to 0.80 as substantial, and above 0.80 as excellent agreement^(13)^. ICC values < 0.50 were considered as poor, from 0.50 to 0.75 as moderate, from 0.75 to 0.90 as good, and > 0.90 as excellent agreement^(14)^.

The questions on the evaluators' opinions underwent descriptive analysis.

## RESULTS

The study included five SLH pathologists with a mean age of 36 years, standard deviation (SD) of 10.6, and a mean experience in facial paralysis rehabilitation of 8.8 years (SD = 3.2). All of them (100%) had a specialist title in Health Professional Residency (Multiprofessional), and two had a master's degree (40%). The 30 patients evaluated had flaccid phase CFP, with a mean of 2.75 days (SD = 1.41) since stroke onset, in the acute phase of the disease.

The interrater agreement results regarding the degree of CFP impairment with the HB scale indicated considerable agreement among most SLH pathologists (kappa between 0.21 and 0.40). Only one rater had moderate agreement, with kappa = 0.41. The interrater agreement results with the SFGS found good agreement among all SLH pathologists, with an ICC value between 0.75 and 0.90 (Table 1).

**Table 1:** Interrater agreement analysis of the interpretation of the degree of central facial paralysis impairment

**Rater**	**HB** *			**SFGS** **		
**1^st^ moment**	**Kappa**	**LL**	**UL**	**ICC**	**LL**	**UL**
**1**	0.321	0.202	0.445	0.817	0.616	0.917
**2**	0.245	0.132	0.356	0.791	0.598	0.883
**3**	0.315	0.232	0.428	0.882	0.558	0.956
**4**	0.414	0.371	0.562	0.807	0.710	0.938
**5**	0.315	0.232	0.428	0.789	0.601	0.894

* Kappa weighted coefficient (K)

** Intraclass correlation coefficient (ICC)

**Caption:** HB: House & Brackmann; SFGS: Sunnybrook Facial Grading System; LL, lower limit of the 95% confidence interval; UL, upper limit of the 95% confidence interval

The intrarater investigation regarding the evaluation and re-evaluation with a 10-day interval found an excellent agreement with the HB scale (kappa = 0.854) and a good agreement with the SFGS (ICC = 0.887) (Table 2).

**Table 2:** Intrarater agreement analysis of the interpretation of the degree of central facial paralysis impairment at the two assessment times

**Assessment**	**HB** *			**SFGS** **		
	**Kappa**	**LL**	**UL**	**ICC**	**LL**	**UL**
**2^nd^ moment**	0.854	0.733	0.929	0.887	0.756	0.956

* Kappa weighted coefficient (K)

** Intraclass correlation coefficient (ICC)

**Caption**: HB: House & Brackmann; SFGS: Sunnybrook Facial Grading System; LL, lower limit of the 95% confidence interval; UL, upper limit of the 95% confidence interval.

The qualitative analysis results showed that all SLH pathologists considered the SFGS the most appropriate scale for assessing and classifying CFP. The following aspects were described as motivations for choosing the scale: it can be used for any phase of facial paralysis (flaccid and sequelae, with or without synkineses); its detailed and quantitative analysis helps measure the evolution of the case based on facial function; it assesses rest, movement, and synkineses/contractures separately; and it is easier to complete than the HB scale, although more laborious.

## DISCUSSION

The results of this study revealed that the SFGS had better interrater agreement than the HB because the latter proved to be more subjective, as it depends on the observation of all segments of the affected hemiface. The agreement was considerable and moderate with the HB scale and good with the SFGS. The intrarater agreement, in turn, was high with both scales, indicating a substantial agreement between the repeated evaluations by the same rater.

Several scales developed to assess PFP are adapted and used to assess and classify CFP. This study chose HB and SFGS mainly because of their different approaches to assessing the patient (respectively general and functional facial aspects), the clinical practices in which they are employed (simple vs. detailed assessment), and how long ago they were developed (old vs. recent publication).

The HB scale was developed in 1985^(4)^ and is the most widely used scale for evaluating and classifying PFP and CFP, despite not being adapted or validated for Brazilian Portuguese^(15)^.

A study in the literature applied this scale for the medical monitoring of patients undergoing surgery for vestibular schwannoma^(16)^. Another study tabulated the evolution of patients with PFP who received physical therapy treatment, based on the evaluation with the HB scale^(17)^. Yet another study used this scale for the epidemiological analysis of patients with CFP in a rehabilitation hospital^(18)^. They demonstrate that different health professionals apply the HB scale, and its simple, objective, categorized form of evaluation is therefore of utmost importance.

The SFGS was translated into Portuguese and adapted to Brazilian culture in 2022 (12). It was developed to provide a reliable and valid method to assess facial function and examine the face separately in specific facial expressions. Moreover, it has a continuous, broad scoring range and is sensitive to small changes in facial movement patterns (7, 12^)^.

A study evaluated the intrarater and interrater agreement with the SFGS among experienced professionals from otorhinolaryngology, physiotherapy, and SLH services in patients with PFP, concluding that it was an easily reproducible scale^(19)^. Van Veen et al.^(20)^ conducted an observational study to analyze the learning curve of inexperienced evaluators using the SFGS in the evaluation of 100 patients with facial paralysis. As a result, the interrater agreement was initially good, but gradually improved over time, stabilizing after approximately 70 evaluations. Thus, the research concluded that the SFGS is a viable evaluation system even for inexperienced evaluators, as long as they receive adequate prior training. This highlights the relevance of the SFGS in clinical practice, as new studies add evidence about its effectiveness in various applications.

An integrative literature review on SLH practice in facial paralysis describes that the main role of these pathologists is to readjust functional aspects of the face, involving speech, chewing, and facial expressions^(21)^. Therefore, SFGS’ better interrater agreement can be explained by its more specific assessment of the thirds of the face and the greater influence of facial functions in its final result when compared to the HB scale.

Few studies have evaluated CFP classification with the agreement methods used here. However, a cross-sectional observational study performed a reliability analysis of the SFGS in 32 patients with subacute stroke and concluded, based on the significant test-retest correlation, that the SFGS is reliable for evaluating facial paralysis also in patients affected by cerebrovascular disease^(22)^.

Mat Lazim et al.^(23)^ conducted a comparative study of three classification systems (HB scale, SFGS, and Sydney Scale) for assessing facial paralysis (regardless of its type) and predicting neural recovery. According to the authors, the HB scale, despite its ease of clinical use, has stopped being used due to insufficient sensitivity to monitor the patients’ clinical evolution. In contrast, the SFGS has advantages by demonstrating high reproducibility of results, also highlighted in the present study and corroborated by the agreement result. Furthermore, according to the referenced research, the SFGS is reliable even for inexperienced evaluators. Neville et al.^(24)^ also identified that professionals unanimously applied the SFGS in cases of facial paralysis.

Several studies in the literature compare different scales applied only to PFP to verify whether they are practical and useful for assessing and classifying facial paralysis in clinical SLH practice. Thus, the present study innovated by analyzing which PFP assessment method would be most appropriate to classify CFP.

Using standardized assessment resources organizes communication between health professionals, directly affecting the order of importance and organization of treatment and the temporal complexity of the process^(25)^. Therefore, the instruments used in clinical and hospital practice should be as specific and reliable as possible.

Further studies are needed on the application of SFGS in patients with CFP, comparing them before and after SLH therapy to analyze its reliability of use and the results in PFP.

Despite this study’s agreement using the SFGS, it is important to emphasize that it was not originally designed to assess or classify CFP^(7)^. Therefore, the need to develop in the medium or long term a specific scale to assess CFP has not yet been ruled out.

## CONCLUSION

This study found that the SFGS performed better in the interrater agreement analysis than the HB scale, although the latter had considerable merits in the intrarater assessment.

Since both presented interrater and intrarater agreements, it is concluded that both scales are suitably adaptable and useful for assessing and classifying CFP. However, the SLH pathologists indicated the SFGS as the most appropriate.

## References

[1] Pimenta E, Costa A, Bule JM, Reis G (2019). Recuperar a expressão facial após parésia facial central. Revista Ibero-Americana de Saúde e Envelhecimento..

[2] Calais LL, Gomez MVSG, Bento RF, Comerlatti LR (2005). Avaliação funcional da mímica na paralisia facial central por acidente cerebrovascular. Pro Fono.

[3] Yoshino Y, Gono Y, Tsuboi K (2024). Acute peripheral facial paralysis caused by tegmental pontine infarction. BMJ Case Rep.

[4] House JW, Brackmann DE (1985). Facial nerve grading system. Otolaryngol Head Neck Surg.

[5] Lacôte M, Chevalier AM, Miranda A, Bleton J, Stevenin P, Lacôte M, Chevalier AM, Miranda A, Bleton JP, Stevenin P (1987). Avaliação clínica da função muscular..

[6] Stennert E, Fish U (1977). Facial Nerve Surgery..

[7] Ross BR, Fradet G, Nedzelski JM (1994). Development of a sensitive clinical facial grading system. Eur Arch Otorhinolaryngol.

[8] Garcia LRS, de Almeida JJ, Souza HAO, Garcia LRS (2021). Acupuntura no tratamento da paralisia facial periférica: uma revisão sistemática. Revista Recien..

[9] Fonseca KMO, Mourão AM, Motta AR, Vicente LC (2015). Scales of degree of facial paralysis: analysis of agreement. Rev Bras Otorrinolaringol (Engl Ed).

[10] Santos JM, da Silva IT (2022). Knowledge of physiotherapists about the treatment of peripheral facial palsy. Res Soc Dev.

[11] Rodríguez-Acelas AL, Cañon-Montañez W (2018). Contribuições das escalas em saúde como ferramentas que influenciam decisões no cuidado dos pacientes. Rev Cuid (Bucaramanga).

[12] Melo TKMD, Andrade PF, Mateus SRM, Santos-Couto-Paz CCD (2022). Psychometric properties of the Brazilian version of the Sunnybrook Facial Grading System. Fisioter Mov.

[13] Landis JR, Koch GG (1977). The measurement of observer agreement for categorical data. Biometrics.

[14] Koo TK, Li MY (2016). A guideline of selecting and reporting intraclass correlation coefficients for reliability research. J Chiropr Med.

[15] Fabricius J, Simple FK, Mohit K (2021). Assessment and rehabilitation interventions for central facial palsy in patients with acquired brain injury: a systematic review. Brain Inj.

[16] Sun MZ, Oh MC, Safaee M, Kaur G, Parsa AT (2012). Neuroanatomical correlation of the House-Brackmann grading system in the microsurgical treatment of vestibular schwannoma. Neurosurg Focus.

[17] Tavares ADC, Souza WP, Jesus EA (2018). Intervenção fisioterapêutica no tratamento de paciente com paralisia facial periférica: estudo de caso. Saúde Pesqui.

[18] Batista KT (2011). Paralisia facial: análise epidemiológica em hospital de reabilitação. Rev Bras Cir Plást.

[19] Cabrol C, Elarouti L, Montava AL, Jarze S, Mancini J, Lavieille JP (2021). Sunnybrook facial grading system: intra-rater and inter-rater variabilities. Otol Neurotol.

[20] Van Veen MM, Bruins TE, Artan M, Werker PMN, Dijkstra PU (2020). Learning curve using the Sunnybrook Facial Grading System in assessing facial palsy: an observational study in 100 patients. Clin Otolaryngol.

[21] Dias MP, Silva MFF, Barreto SS (2021). Reabilitação fonoaudiológica na paralisia facial periférica: revisão integrativa. Audiol Commun Res.

[22] Tramontano M, Morone G, LA Greca FM, Marchegiani V, Palomba A, Iosa M (2021). Sunnybrook Facial Grading System reliability in subacute stroke patients. Eur J Phys Rehabil Med.

[23] Mat Lazim N, Ismail H, Abdul Halim S, Nik Othman NA, Haron A (2023). Comparison of 3 Grading Systems (House-Brackmann, Sunnybrook, Sydney) for the assessment of facial nerve paralysis and prediction of neural recovery. Medeni Med J..

[24] Neville C, Beurskens C, Diels J, MacDowell S, Rankin S (2024). Consensus among international facial therapy experts for the management of adults with unilateral facial palsy: a two-stage nominal group and Delphi study. Facial Plastic Surgery Aesthetic Medicine.

[25] Gardona RGB, Barbosa DA (2018). The importance of clinical practice supported by health assessment tools. Rev Bras Enferm.

