# Supplementary material for: Classification of central facial paralysis: an agreement analysis
Source: Codas. 2025 May 2;37(3):e20240158. doi: 10.1590/2317-1782/e20240158en (PMC12061339; doi:10.1590/2317-1782/e20240158en)
Supplement: Translation

# Translation: Classificação da paralisia facial central: análise de concordância

**DOI:** 10.1590/2317-1782/e20240158pt

**Authors:** Nathallie Angel Conceição da Silva Andrade, Raquel Karoline Gonçalves Amaral, Laélia Cristina Caseiro Vicente, Aline Mansueto Mourão

**Affiliations:** 1 Departamento de Fonoaudiologia, Universidade Federal de Minas Gerais – UFMG - Belo Horizonte (MG), Brasil.

**Keywords:** Paralisia Facial, Avaliação, Escalas, Classificação, Fonoaudiologia

## Abstract

**Objetivo:**

Identificar se há concordância inter e intra-avaliadores na classificação do grau de paralisia facial central por meio de duas escalas, e qual delas é mais adequada para classificar a gravidade da mímica facial de acordo com a opinião de especialistas.

**Método:**

Estudo observacional, prospectivo de corte transversal de análise de concordância das escalas de House & Brackmann (HB) e do Sistema de Graduação Facial Sunnybrook (SFGS), realizado com cinco fonoaudiólogos com experiência clínica que analisaram a expressão facial de 30 indivíduos adultos após Acidente Vascular Cerebral para concordância interavaliadores. As avaliações foram realizadas em duas etapas, com 10 dias de intervalo, sendo a segunda etapa com 20% da amostra inicial para concordância intra-avaliadores. Foi empregado o coeficiente kappa ponderado para a escala HB e o coeficiente de correlação intraclasse ICC para a classificação SFGS.

**Resultados:**

A escala HB indicou concordâncias interavaliadores considerável e intra-avaliadores excelente. O SFGS apresentou boa concordância interavaliadores e intra-avaliadores. Todos os fonoaudiólogos consideraram o SFGS como a escala mais adequada para classificação da paralisia facial central.

**Conclusão:**

O SFGS demonstrou desempenho superior na análise da concordância interavaliadores. A escala HB evidenciou méritos consideráveis na avaliação intra-avaliadores. Ambas as escalas estão aptas a serem adaptadas e utilizadas na avaliação e classificação da paralisia facial central, contudo, os fonoaudiólogos indicaram a SFGS como a mais adequada.

## INTRODUÇÃO

A paralisia facial central (PFC) é uma das consequências mais comuns em pacientes após o Acidente Vascular Cerebral (AVC), acometendo mais da metade desses indivíduos^(1)^. Esse acometimento se manifesta por assimetria facial e incapacidade muscular principalmente para a mímica facial no terço inferior da face, influenciando na funcionalidade e nos aspectos estéticos, sociais e emocionais do paciente^(1,2)^.

A PFC se difere da paralisia facial periférica (PFP), sendo as principais diferenças relacionadas à localização da lesão, tipo de movimentos afetados e terço facial acometido. Dessa forma, a PFP caracteriza-se por acometimento de toda a hemiface dos movimentos voluntários e involuntários, ipsilateral à lesão, e é ocasionada por lesões do núcleo do nervo facial (nucleares) ou do nervo facial (inervação infranuclear). Por outro lado, na PFC, a lesão ocorre acima do núcleo do nervo facial no tronco cerebral, geralmente devido a lesões ou disfunções no córtex cerebral, na via corticobulbar ou nas áreas adjacentes. Nesse caso, a musculatura voluntária do terço inferior e médio da face, contralateral à lesão, é acometida, devido ao núcleo do nervo facial que inerva a hemiface inferior e recebe fibras corticonucleares (inervação supranuclear) do hemisfério contralateral. Já o núcleo do facial que inerva o segmento superior da face recebe fibras corticonucleares dos dois hemisférios cerebrais, sendo preservada a mímica da hemiface superior em lesões do trato corticonuclear^(2)^.

É importante ressaltar que a PFC resulta geralmente de um AVC na artéria cerebral média ou seus ramos. No entanto, AVC em outras artérias, como a basilar ou a meníngea média, também pode causar PFP, pois contribuem para a irrigação do nervo facial^(3)^.

Na literatura são diversos os protocolos existentes para a classificação da PFP, incluindo as escalas de House & Brackmann ^(4)^, Chevalier (1987)^(5)^, Yahanagihara (1976)^(6)^ e o Sistema de Graduação Facial Sunnybrook (SGFS) (1994)^(7)^. Porém, durante pesquisas, não foi possível encontrar escalas exclusivas para a classificação das expressões faciais nos casos de PFC. A escala de House & Brackmann avalia os três terços da face em repouso e em movimento e sua classificação é constituída em seis graus e, de acordo com a literatura, é a mais utilizada nos dois tipos de paralisia, tanto clinicamente quanto em pesquisas científicas^(8)^, sendo adaptada aos casos de PFC^(9)^. O SGFS analisa a face em três parâmetros clínicos: simetria em repouso, simetria nos movimentos e a ocorrência de contraturas e sincinesias e tem como objetivo principal a classificação da paralisia pautada na funcionalidade facial^(7)^.

Na vivência dos profissionais de saúde, a avaliação é um fator primordial e consiste em identificar e definir as condições clínicas do paciente e os recursos disponíveis para determinar a intervenção apropriada para a performance funcional^(10)^. A capacidade de analisar corretamente o movimento alterado é um pré-requisito essencial para avaliação de pacientes com comprometimento funcional desse movimento^(10)^.

Dito isso, a utilização de escalas específicas para mensuração do grau de PFC pode ter grande impacto no que diz respeito à priorização de intervenções e acompanhamento da evolução dos pacientes^(11)^. É de suma importância, na prática clínica fonoaudiológica, a referência de escalas com alto grau de confiabilidade e fidedignidade, de forma a favorecer a qualidade do cuidado e otimizar o tempo dos profissionais^(11)^. Assim sendo, a PFC pode requerer uma avaliação mais direcionada, exigindo do profissional um olhar específico para os movimentos alterados que comprometem a mímica facial. Por isso, verificar se as escalas validadas para PFP podem ser aplicados para PFC faz-se importante para a prática clínica fonoaudiológica, uma vez que corrobora ao correto diagnóstico e viável intervenção pautada na performance funcional do indivíduo.

Dessa forma, o presente estudo teve como objetivos: 1) identificar se há concordância inter e intra-avaliadores na classificação do grau de PFC, por meio de duas escalas, House & Brackmann e o Sistema de Graduação Facial Sunnybrook, e 2) e identificar se alguma dessas escalas pode ser mais adequada para PFC de acordo com a opinião de especialistas.

## MÉTODO

Trata-se de um estudo observacional transversal aprovado pelo Comitê de Ética e Pesquisa da Universidade Federal de Minas Gerais sob o parecer 5.019.519. Para a realização do estudo, cinco fonoaudiólogas com experiência em reabilitação de paralisia facial foram convidadas a avaliar 30 casos clínicos de pacientes com PFC após AVC. As profissionais concordaram em participar da pesquisa e assinaram o termo de consentimento livre e esclarecido.

Para tanto, foram utilizadas fotografias e filmagens, oriundas do banco de dados dos atendimentos de pacientes com PFC pós-AVC da Unidade de Acidente Vascular Cerebral do Hospital Risoleta Tolentino Neves apresentando as seguintes expressões faciais: “em repouso”, “cara de assustado”, “cara de bravo”, “fechar os olhos suavemente”, “fechar os olhos com força”, “sorriso aberto”, “sorriso fechado”, “cara de triste”, “cara de cheiro ruim”, “beiço” e “bico”.

As cinco fonoaudiólogas avaliaram as fotografias e filmagens de cada paciente a partir de duas escalas diferentes: “House & Brackmann - HB” (1985)^(4)^, a qual não foi adaptada e validada para o português brasileiro e “Sistema de Graduação Facial Sunnybrook - SFGS” (1994)^(7)^, traduzido, adaptado e validado para o português brasileiro no ano de 2022^(12)^.

Na escala HB (Quadro 1), a avaliação da mímica facial ocorre tanto em repouso quanto em movimento para as expressões faciais, classificando a paralisia facial pelo nível de acometimento da paralisia pelos terços da face, em conjunto, graduada em seis níveis^(4)^. A escala HB adaptada para avaliação dos pacientes com PFC consiste: em repouso, avaliação da simetria facial, analisando os três terços da face e, em movimento, apenas o terço inferior da face^(4)^.

**Quadro 1:** Avaliação de movimentação facial segundo House & Brackmann (1985)

**Grau**	**Descrição**	**Em repouso**	**Em Movimento**
I **()**	Normal	Simetria	Função facial normal
II **()**	Disfunção Leve	Simetria e tônus normais	*Fronte*: função moderada a boa
			*Olho*: fechamento completo com esforço mínimo
			*Boca*: assimetria discrete
III **()**	Disfunção moderada	Simetria e tônus normais	*Fronte*: movimento discreto a moderado
			*Olho*: fechamento completo com esforço
			*Boca*: discreta fraqueza com máximo esforço
IV **()**	Disfunção moderadamente severa	Simetria e tônus normais	*Fronte*: nenhum
			*Olho*: fechamento incompleto
			*Boca*: assimetria com esforço máximo
V **()**	Disfunção severa	Assimetria	*Fronte*: nenhum
			*Olho*: fechamento incompleto
			*Boca*: discreto movimento
VI **()**	Paralisia total	Assimetria	Nenhum movimento

O SFGS (Figura 1), além de avaliar a mímica facial em relação à simetria em repouso e à simetria dos movimentos voluntários, leva em consideração as sincinesias associadas aos movimentos testados^(7,12)^. As principais diferenças entre as escalas são que o SFGS oferece um resultado numérico mais amplo e detalhado, considerando a função de músculos faciais específicos, bem como a presença ou não de sincinesia.

**Figura 1:** Sistema de Graduação Facial Sunnybrook (2022)

Inicialmente, as fonoaudiólogas participantes do estudo realizaram treinamento online prévio, que teve como objetivo fornecer base técnica padrão para a avaliação da paralisia facial por meio das duas escalas, uma vez que as escalas HB e SFGS não eram de uso habitual dos profissionais convidados, embora possuíssem experiência no acompanhamento de pacientes com paralisia facial. Assim, o treinamento foi para apresentar as escalas e esclarecer sobre as formas de avaliações, já que elas não são específicas para PFC e poderiam ser utilizadas de maneiras diferentes pelos profissionais, o que inviabilizaria a análise de concordância dos resultados e consequentemente, o estudo. Portanto, o treinamento visou instrumentalizar os profissionais em relação ao modo de uso das escalas e não em como interpretar a mímica facial.

Para o treinamento foram utilizados três casos de paralisia facial central distintos dos 30 casos apresentados para análise propriamente dita. Os 30 casos clínicos foram selecionados a partir do banco de dados de fotos e filmagens por meio de amostragem estratificada de acordo com a classificação de HB realizada por uma fonoaudióloga experiente para equilíbrio entre os níveis de gravidade das paralisias.

Após o treinamento, no intuito de obter-se a concordância interavaliadores, as fonoaudiólogas receberam as fotografias e os filmagens dos 30 casos clínicos, e, avaliaram os casos por quantas vezes julgasse necessário, dentro do prazo de 20 dias, de acordo com as duas escalas (HB e SFGS) sem a possibilidade de discussão entre pares.

Após a entrega da primeira avaliação e com o intervalo de 10 dias, iniciou-se o processo de pesquisa da concordância intra-avaliadores por meio da segunda avaliação. Para tanto, as cinco fonoaudiólogas receberam uma amostra de 20% da amostra inicial (seis casos), categorizados de forma diferente da primeira avaliação, para que os avaliassem novamente por meio dos mesmos critérios estabelecidos na primeira avaliação, também de acordo com as duas escalas.

Além da avaliação dos casos, as fonoaudiólogas foram solicitadas a responder o Questionário de Preferência da Classificação da Paralisia Facial (Quadro 2), o qual foi elaborado pelas próprias autoras, com duas perguntas, a fim de elencar a melhor escala para o objetivo proposto e explicitar os motivos para a escolha, respectivamente.

**Quadro 2:** Questionário de Preferência da Classificação da Paralisia Facial

1. Qual escala seria mais adequada para avaliação da paralisia facial central?
() House & Brackmann (HB)
() Sistema de Graduação Facial Sunnybrook (SFGS)
2. Qual(is) o(s) motivo(s) para a sua escolha?

As respostas das fonoaudiólogas foram tabuladas e os resultados submetidos à análise estatística por meio do software SPSS versão 20. Para realizar as análises de concordância inter e intra-avaliadores foram utilizados o coeficiente kappa ponderado (K) para a classificação HB por ser categórica ordinal e o coeficiente de correlação intraclasse (ICC) para a classificação SFGS por ser mensurada como variável contínua. As análises permitiram medir a concordância entre dois ou mais avaliadores^(13,14)^. Para a verificação da concordância intra-avaliadores levou-se em consideração a primeira e a segunda avaliação com reprodutibilidade de 20% da amostra total.

Foram considerados os valores de kappa igual a 0 como pobre concordância, de 0 a 0,20 ligeira, de 0,21 a 0,40 considerável, entre 0,41 e 0,60 como moderada, de 0,61 a 0,80 substancial, e acima de 0,80 excelente concordância^(13)^. Para os valores de ICC, < 0,50 foram considerados como pobre, entre 0,50 e 0,75 moderada, 0,75 e 0,90 boa e > 0,90 excelente^(14)^.

Para as questões relacionadas à opinião dos avaliadores, foi realizada análise descritiva.

## RESULTADOS

Participaram do estudo 5 fonoaudiólogas com idade média de 36 anos, desvio padrão (DP) de 10,6, e tempo médio de experiência em reabilitação de paralisia facial de 8,8 anos (DP=3,2), sendo que todas (100%) possuem o título de especialista na modalidade Residência em Área Profissional da Saúde (Multiprofissional) e 2 fonoaudiólogas com o título de mestre (40%). Os 30 pacientes avaliados apresentavam PFC em fase flácida com tempo de ictus médio de 2,75 dias (DP=1,41) de AVC na fase aguda da doença.

Os resultados da avaliação da concordância interavaliadores na interpretação do grau de comprometimento da PFC para a escala HB indicaram concordância considerável na maioria dos fonoaudiólogos (kappa entre 0,21 e 0,40). Apenas um avaliador apresentou moderada concordância, ao apresentar kappa = 0,41. Ao analisar os resultados da concordância interavaliadores para o SFGS, houve boa concordância entre todos os fonoaudiólogos com valor ICC entre 0,75 e 0,90 (Tabela 1).

**Tabela 1:** Avaliação da concordância interavaliadores na interpretação do grau de comprometimento da paralisia facial central

**Avaliador**	**HB** *			**SFGS** **		
**1º momento**	**Valor Kappa**	**LI**	**LS**	**Valor ICC**	**LI**	**LS**
**1**	0,321	0,202	0,445	0,817	0,616	0,917
**2**	0,245	0,132	0,356	0,791	0,598	0,883
**3**	0,315	0,232	0,428	0,882	0,558	0,956
**4**	0,414	0,371	0,562	0,807	0,710	0,938
**5**	0,315	0,232	0,428	0,789	0,601	0,894

* Coeficiente Kappa ponderado (K)

** Coeficiente de correlação intraclasse (ICC)

**Legenda:** HB: House & Brackmann; SFGS: Sistema de Graduação Facial Sunnybrook; LI, limite inferior do intervalo de 95% de confiança; LS, limite superior do intervalo de 95% de confiança

Na investigação intra-avaliadores, em relação à avaliação e reavaliação com dez dias de intervalo entre as mesmas, na escala HB houve excelente concordância com kappa = 0,854. No SFGS, houve boa concordância com ICC = 0,887 (Tabela 2).

**Tabela 2:** Avaliação da concordância intra-avaliadores na interpretação do grau de comprometimento da paralisia facial central nos dois momentos de avaliação

**Avaliação**	**HB** *			**SFGS** **		
	**Valor Kappa**	**LI**	**LS**	**Valor ICC**	**LI**	**LS**
**2° momento**	0,854	0,733	0,929	0,887	0,756	0,956

* Coeficiente Kappa ponderado (K)

** Coeficiente de correlação intraclasse (ICC)

**Legenda:** HB: House & Brackmann; SFGS: Sistema de Graduação Facial Sunnybrook; LI, limite inferior do intervalo de 95% de confiança; LS, limite superior do intervalo de 95% de confiança.

Quanto à análise qualitativa, o resultado demonstrou que todos os fonoaudiólogos consideraram o SFGS como a escala mais adequada para avaliação e classificação da PFC. Como motivações para a escolha foram descritos os seguintes aspectos: é possível ser usado para qualquer fase da paralisia facial (flácida e de sequela com presença ou não de sincinesias); possui uma análise detalhada e quantitativa que facilita a mensuração da evolução do caso a partir da função facial; avalia os aspectos repouso, movimento e presença de sincinesias/contraturas separadamente; e apresenta facilidade de preenchimento, apesar de mais trabalhosa, quando comparada à escala HB.

## DISCUSSÃO

Os resultados do presente estudo revelaram que, no que diz respeito à concordância interavaliadores, o SFGS demonstrou melhor concordância em comparação com a HB. Isso porque a escala HB se mostrou mais subjetiva, pois depende da observação de todos os segmentos de hemiface afetada. Foi possível observar níveis de concordância consideráveis e moderados na escala HB, enquanto a concordância no SFGS foi avaliada como boa. Ao examinar a concordância intra-avaliadores, observa-se um alto grau de concordância em ambas as escalas, indicando uma concordância substancial entre as avaliações repetidas pelo mesmo avaliador.

Dentre as escalas existentes para a avaliação de paralisia facial periférica que são adaptadas e utilizadas em avaliações e classificações de paralisia facial central, a escolha da HB e do SGFS para a realização do presente estudo pautou-se, principalmente, nas diferentes abordagens que as escalas utilizam para avaliar o paciente (aspecto facial: geral e funcional, respectivamente), assim como nas práticas clínicas a que são empregadas (avaliação: simples *versus* detalhada) e há quanto tempo foram desenvolvidas (ano de publicação: antiga *versus* atual).

A escala HB foi desenvolvida em 1985^(4)^ e, apesar de não ser adaptada e validada para o português brasileiro, é a escala mais utilizada para avaliação e classificação de paralisias faciais, periféricas ou centrais^(15)^.

Na literatura, um estudo aplicou essa escala com o intuito de acompanhamento médico de pacientes submetidos a cirurgia de schwannoma vestibular^(16)^. Outro estudo realizou a tabulação, a partir da avaliação pela escala HB, da evolução de pacientes acometidos por PFP que receberam tratamento fisioterápico^(17)^. Outra pesquisa utilizou essa escala para análise epidemiológica de pacientes com PFC em um hospital de reabilitação^(18)^. Assim, percebe-se que a escala HB é aplicada por diferentes profissionais da área da saúde, sendo de suma importância, então, sua forma de avaliação simples, objetiva e categorizada.

O SGFS foi traduzido e adaptado para a Língua Portuguesa e cultura brasileira no ano de 2022^(12)^, e o mesmo foi desenvolvido com a finalidade de oferecer um método confiável e válido para avaliar a função facial, de forma a examinar a face separadamente em expressões faciais específicas. Além disso, propõe uma faixa de pontuação contínua e ampla, sensível a pequenas mudanças nos padrões de movimentos faciais^(7,12)^.

Um estudo avaliou a concordância intra e interavaliadores do SFGS com profissionais experientes dos serviços de otorrinolaringologia, fisioterapia e fonoaudiologia em pacientes com PFP, concluindo-a como uma escala facilmente reprodutível^(19)^. Van Veen et al.^(20)^ realizaram um estudo observacional para analisar a curva de aprendizado de avaliadores inexperientes utilizando-se o SFGS na avaliação de 100 pacientes com paralisia facial. Como resultado, a concordância interavaliadores foi inicialmente boa, mas melhorou gradativamente ao longo do tempo, estabilizando-se após aproximadamente 70 avaliações. Com isso, a pesquisa concluiu que o SFGS é um sistema de avaliação viável mesmo para avaliadores inexperientes, desde que os mesmos recebam adequado treinamento prévio. Dessa forma, torna-se exposta a relevância do SFGS na prática clínica, à medida que novas pesquisas acumulam evidências acerca da sua eficácia em diversas aplicações.

Uma revisão de literatura integrativa acerca da atuação fonoaudiológica na paralisia facial descreve como papel principal do fonoaudiólogo a readequação dos aspectos funcionais da face, envolvendo fala, mastigação e expressões faciais^(21)^. Assim sendo, a superior concordância interavaliadores do SFGS pode ser explicada por sua avaliação mais específica dos terços da face, além de apresentar maior influência das funções faciais em seu resultado final quando comparada à escala HB.

São escassos os estudos de avaliação de classificação de PFC com os métodos de concordância aqui utilizados, porém um estudo observacional transversal realizou a análise de confiabilidade do SFGS em 32 pacientes com AVC subagudo e concluiu, a partir da correlação significativa entre teste e reteste, que o SFGS é confiável para avaliar a paralisia facial também em pacientes acometidos por doença cerebrovascular^(22)^.

Mat Lazim et al.^(23)^ realizaram um estudo comparativo de três sistemas de classificação para avaliação de paralisia facial, independente do tipo de paralisia, e previsão da recuperação neural: escala HB, SGFS e Escala de Sydney. De acordo com os autores, a escala HB, apesar da facilidade no uso clínico, tem deixado de ser adotada devido à insuficiência de sensibilidade em relação ao acompanhamento da evolução clínica dos pacientes. Em contrapartida, o SFGS apresenta vantagens por demonstrar alta reprodutibilidade de resultados, que também foram destacados no presente estudo e corroborados pelo resultado da concordância. Além disso, de acordo com a pesquisa referenciada, o SFGS mantém-se confiável mesmo para avaliadores inexperientes. Ademais, Neville et al.^(24)^ identificaram a aplicação da SFGS na paralisia facial em posição unânime entre os profissionais.

Na intenção de verificar a praticidade e utilidade das escalas de avaliação e classificação da paralisia facial na prática clínica fonoaudiológica, encontra-se, na literatura, diversas pesquisas de comparação de diferentes escalas aplicadas somente à PFP. Assim, como inovação, o presente estudo interessou-se em analisar qual método de avaliação de PFP seria mais adequado para a classificação de PFC.

A utilização de recursos padronizados de avaliação organiza a comunicação entre os profissionais de saúde, o que afeta diretamente a ordem de importância e organização do tratamento, bem como a complexidade temporal envolvida no processo^(25)^. Assim, espera-se que os instrumentos utilizados na prática clínica e hospitalar sejam os mais específicos e confiáveis possíveis.

Portanto, faz-se necessários mais estudos acerca da aplicação do SGFS em pacientes com PFC, comparando momentos pré e pós terapia fonoaudiológica, a fim de analisar a sua confiabilidade de uso além dos resultados obtidos em paralisias faciais periféricas.

Embora a presente escala tenha demonstrado concordância nesse estudo, é importante ressaltar que ela não foi originalmente concebida para a avaliação e classificação de paralisias faciais centrais^(7)^. Portanto, no horizonte de médio e longo prazo, a necessidade de desenvolver uma escala específica destinada à avaliação de paralisias faciais centrais ainda não foi descartada.

## CONCLUSÃO

Observou-se que o SGFS demonstrou um desempenho superior na análise da concordância interavaliadores quando comparada à escala HB, embora esta última tenha evidenciado méritos consideráveis na avaliação intra-avaliadores.

Visto que ambas apresentaram concordâncias inter e intra-avaliadores, conclui-se que ambas as escalas estão aptas a serem adaptadas e utilizadas na avaliação e classificação da paralisia facial central, contudo, os fonoaudiólogos indicaram a SFGS como a mais adequada.
